# Expression correlates between Annexin A1 and A2 autoantibodies in patients with Behçet’s disease

**DOI:** 10.1042/BSR20170778

**Published:** 2018-01-19

**Authors:** Muhammad Hussain, Peng Chen, Guang Mei, Yongzhe Li, Hongwu Du

**Affiliations:** 1 112 Lab, School of Chemistry and Biological Engineering, University of Science and Technology Beijing, Beijing 100083, China; 2 Department of Rheumatology and Clinical Immunology, Peking Union Medical College Hospital, Chinese Academy of Medical Sciences and Peking Union Medical College, Key Laboratory of Rheumatology and Clinical Immunology, Ministry of Education, Beijing, China

**Keywords:** Annexin A1, Annexin A2, Autoimmunity, Chinese Hans population, Immunogenicity

## Abstract

The autoantibodies profile of Behçet’s disease (BD) is yet incompletely understood. Annexins are a family of highly conserved proteins which are involved in some human autoimmune diseases. Autoantibodies directed toward Annexin A1 and A2 are involved in BD pathology, but correlation in their clinical role is controversial. The aim of our study is to estimate and evaluate the expression correlation between Annexin A1 and A2 autoantibodies in BD patients. We have designed and implemented different technical approaches to prove the hypothesis. First, bioinformatics tools such as amino acid sequence alignment, epitope prediction analysis, and 3D structural comparison were performed to find out the correlation between Annexin A1 and A2. Second, amplification of the corresponding gene by RT-PCR, then cloning, and purification techniques were applied to acquire the recombinant Annexin A1. Third, the target protein band was excised from gel electrophoresis, digested with trypsin, and analyzed by MALDI-TOF/TOF. Finally, in-house ELISA was developed to determine the induced anti-Annexin A1 autoantibodies in BD patients. Obtained results demonstrated that the BD serum reactivity against recombinant Annexin A1 was significantly higher as compared with healthy control (HC) (*P*<0.001). Moreover, bioassay results of Annexin A1 and A2 also showed the presence, absence, and independent coexistence of autoantibodies, when reacted with BD sera. In conclusion, Annexin A1 has a similar immunogenic expression and correlation with its analog Annexin A2 and their association may be a novel immune target of BD in Han Chinese population.

## Introduction

Behçet’s disease (BD) is a rare, chronic, and autoinflammatory condition of unknown origin. It is caused by vasculitis that can damage blood arteries and veins throughout the body. It has multisystemic triplet symptoms disease of oral, genital ulcer, and ocular inflammation [1]. The disease was named in 1937, after its first identifier Hulusi Behçet. Geographically, BD is distributed all over the world but more prevalent along the old Silk Road, extending from Middle East to China [2,3]. BD has a variety of clinical expressions, indicating the co-existence in their autoantigens [4,5]. Similarly, it has been reported that BD has an association with various autoimmune and inflammatory disorders such as polymyositis, Sjögren syndrome (SS), Crohn’s disease (CD), systemic lupus erythematosus (SLE), and rheumatoid arthritis (RA) [6,7]. Endothelial cells dysfunction [8,9] and vasculitis [10,11] are major pathological findings in BD, although the etiopathogenesis of the disease is still obscure. Anti-endothelial cell antibodies (AECAs) play a part in pathology of vascular injury and vasculitis which is the main cause of autoimmune diseases [12,13].

Evidences have been given by different studies that AECA in BD patients play a key role as an autoantigen in human dermal microvascular endothelial cells [14,15]. New biomarkers of AECA in BD like HSP27 and prohibitin have been successfully identified by our team [16,17]. Altogether these findings added new information in understanding the AECA pathology and further confirmed the key role of AECA in BD. It is still believed that the cell membrane proteins are involved in pathogenesis of autoimmune diseases. Recently, we had identified the presence of Annexin A2 (36 kDa) molecule on cell membrane surface and it might act as immune target of BD. Immunohistochemistry (IHC) showed high expression of Annexin A2 in vascular endothelial cells and is found approximately in one-third Han Chinese BD patient’s blood circulation. Furthermore, vascular involvement in anti-Annexin A2 positive group was higher than anti-Annexin A2 negative group, mentioning that Annexin A2 is a potential autoantigen of endothelial cell membrane and is involved in BD pathology [18]. On the other hand, Annexin A1 may have a probable anti-inflammatory role in which glucocorticoids regulate the expression and function of Annexin A1 [19]. Anti-Annexin A1 antibodies were detected in multiple autoimmune diseases [20,21].

The present study is designed to evaluate and assess the expression correlation between Annexin A1 and A2 autoantibodies in BD patients. The results obtained from sequence alignment, 3D structural comparison, MS and ELISA have confirmed that Annexin A1 might be involved in BD pathology.

## Materials and methods

### Collection of serum samples

One hundred and seventy-six subjects were enrolled in the present study. Samples were categorized into four groups. (i) BD: an experimental group (*n*=44) with average age of 39 years and age range of 16–62, (ii) SLE: diseased control group (*n*=44) with average age of 31 years and age range of 18–43, (iii) RA: diseased control group (*n*=44) with average of 45 years and age range of 34–83, (iv) HC: healthy control (HC) group (*n*=44) with average of 25 years and age range of 25–38. BD diagnosis was carried out in accordance with the International criteria (International Study Group for BD, 1990). Peking Union Medical College Hospital Ethical Committee approved the sample collection and usage of human specimens from consenting patients. Serum samples were aliquoted and kept at −80°C until used.

### Cell lines

An appropriate human umbilical vein endothelial cell (HUVEC) lines were commercially purchased from American Type Culture Collection (ATCC). Its integrity and purity were tested carefully before the experiment. Culturing conditions were set as per our previous methods [22]. Cells were mixed with TRIzol reagents (Invitrogen, CA), centrifuged, and RNA was extracted.

### Sequence alignment and antigenic epitope prediction

Sequence alignment was performed with protein of known structural homology, i.e. Annexin A2 and co-ordinates were taken from Immune Epitope Database (IEDB). Clustal X software (EMBL, Heidelberg, Germany) was used with its default parameters (Gonnet 250 protein weight matrix) [23]. Then epitopes of two proteins were predicted by Bepipred Linear Epitope Prediction method [24]. In this method, probable similar epitopes from both proteins were selected with the standard that lengths of amino acids were not less than eight and were concomitant of each other. Then, 3D structural similarity analysis was performed using flexible structural alignment method (flexible structure alignment by chaining aligned fragment pairs allowing twist (FATCAT)) [25,26].

### PCR amplification, cloning, expression, and purification of Annexin A1

This method was performed as per our standard lab protocol [27]. In this procedure, mRNA was extracted from HUVECs. Forward and reverse primers were designed as mentioned below and were used to amplify the target gene (*Annexin A1*). RT-PCR was carried out as per kit’s instructions (Fermentas, MD).

Forward: (BamHI) 5′-CGCGGATCCGCGGAAGAGCAGGAATATGTT-3′ (upstream)

Reverse: (XhoI) 5′CCGCTCGAGCGGTTATTATCCACAAAGAGCCACC-3′ (downstream)

After enzyme digestion, the PCR product encoding *Annexin A1* gene was ligated with a plasmid expression vector pET-28(a^+^), then transformed into *Escherichia coli* DH5α and recovered in Super Optimal Broth (SOC) medium. Then the same was transferred to LB medium containing antibiotic kanamycin (50 μl/ml). Extracted recombinant plasmid from *E. coli* DH5α was transformed into the high expression engineering strain *E. coli* BL21. Optimal expression of recombinant *Annexin A1* gene was achieved by adding the IPTG (1 mM) when the level of optical density (OD) reaches 0.4–0.6. Finally, the recombinant Annexin A1 with N-terminal hexahistidine-tag was purified by Ni-NTA resin kit (Qiagen, Hilden, Germany), as described by our recent studies [15], then concentration of recombinant Annexin A1 protein was measured by kit (Biosynthesis Biotechnology, Beijing, China) and stored at −80°C for further testing.

### Identification of Annexin A1 by MS

This method was applied in our recent studies [28]. Briefly, the target protein (recombinant Annexin A1) band was cutoff from SDS/PAGE gel. Then destained the excised target gel pieces with a mixture of 25 mM NH_4_HCO_3_ and 50% acetonitrile, the mixture was dehydrated in a vacuum centrifugation then soaked in a 10 mM dithiothreitol (DDT) and 25 mM NH_4_HCO_3_ for almost 2 h at 37°C. Then added equal volume of 25 mM NH_4_HCO_3_ and 55 mM iodoacetamide to replace the DTT solution and incubated at room temperature for 45 min in dark. The gel pieces were washed with 25 mM NH_4_HCO_3_ and 50% acetonitrile for 10 min. Then digested the dried target gel pieces by mixing 20 μl (0.05 mM NH_4_HCO_3)_ buffer-containing trypsin (Sigma, MO) for overnight at 37°C. Finally, the target protein was identified by proteomics analyzer LC-MALDI-TOF/TOF mass spectrometer (4700, Applied Biosystems, Foster City, CA) and the MS data were analyzed with Mascot Bioinformatics Database Software (Matrix Sciences, London, U.K.); www.matrixscience.com.

### In-house developed ELISA

Microtiter plates 96-wells (Corning, NY) were coated whole night at 4°C with purified recombinant Annexin A1. Then coated plates were washed with PBS solution with Tween-20 (PBST) and blocked the wells with 200 µl goat serum (5%) and incubated for 1 h at 37°C. Dispense the blocking serum from the plate and added patients serum samples in dilution buffer with the ratio 1:100 followed by 2 h incubation at temperature 37°C. Again plates were washed with PBST three times and then added 100-µl antihuman conjugated HRP/IgM (ImmunoHunt, Beijing, China) to each well and incubated again for an additional 1 h at temperature 37°C. Dispense the liquid and added 100 μl TMB substrate solution and incubated at room temperature for 10 min in a dark place. Finally, added 50 μl (2 M H_2_SO_4_, stop solution) and measured the absorbance with the plate reader (Tecan, Hombrechtikon, Switzerland) at 450/620 nm.

### Statistical procedures

Data were evaluated by Fisher test and Wilcox test by using SPSS (version 21, Chicago, IL). *P*<0.05 was taken as a standard and was considered significant. Number with higher value than HC was defined as critical point (mean + 2 S.D.). Then statistical software MedCalc (version 9.2) was used to diagnose healthy/diseased groups. Sensitivity (positive rate) and (100-specificity) means false positive rates were plotted in the form of receiver operating characteristic (ROC) curve. Moreover amino acid sequence data,Mass spectrometry results and chromatogram of pET 28a-Annexin A1 are shown in supplementary materials.

## Results

### Sequence alignment

**Figure 1:**
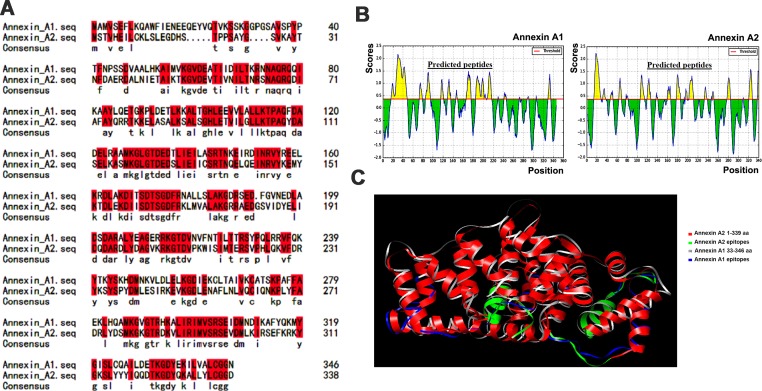
Amino acid sequence alignment, antigenic epitope prediction analysis, and 3D structural comparison between Annexin A1 and A2 (**A**) Sequence alignment results of two human proteins Annexin A1 and A2 (identical amino acids are marked in red color while black color have similar properties). (**B**) Antigenic epitope prediction analysis. (A) Antigenic epitopes distribution of Annexin A1 and A2. The X-axis and Y-axis represent the position and scores, respectively (threshold =0.35). The regions having β turns are shown in yellow. The highest peak region indicates the most potent antigenic epitope. (**C**) 3D structural comparison between Annexin A1 and A2 using homology protein modeling, Annexin A1 (33–346 aa), Annexin A2 (1–339 aa). Similarly, blue color lines indicate Annexin A1 amino acid position and green color lines demonstrate Annexin A2 amino acid position.

Amino acid sequence alignment of recombinant Annexin A1 and Annexin A2 was performed as stated in methodology part. Sequence similarity between two proteins were approximately 55% which indicate that both proteins have evolutionary relationship and certain numbers of the amino acid residues in both proteins have conserved regions. Sequences were identified between target and template proteins. The identical amino acids were shown in red lines while similar amino acid residues are shown in black color as illustrated in Figure 1A. Further, Bepipred Linear Epitope Prediction analysis between Annexin A1 and A2 revealed that the locations of continuous epitopes have been predicted based on Emini’s surface accessibility scale (threshold setting =0.35). Epitopes were predicted in yellow color graph when peptide residue score was above the threshold but not green as shown in Figure 1B, in this figure X-axis shows the position whereas Y-axis represents residue scores. Then 3D structure was obtained from Data Bank database (http://www.rcsb.org) having structural similarity as (Annexin A1: 33–346 aa; Annexin A2: 1–339 aa) as depicted in Figure 1C. The significance of the similarity was detected by FATCAT and *P*-value showed the chance of getting the same similarity in two random structures. Both proteins showed similar structural pairing with probability *P*<0.05 with raw score (score =927.07), indicating that Annexin A1 and Annexin A2 have a close similarity in their structural level and reveal that they have similar antigenic worth.

### Cloning and preparation of recombinant Annexin A1

**Figure 2:**
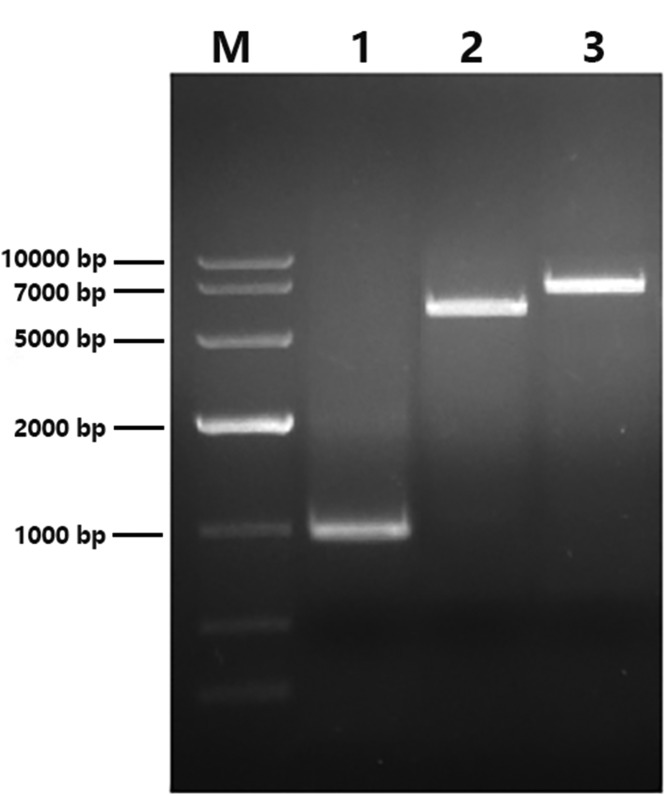
Gene amplification, cloning, and expression M, DNA marker; lane 1, Annexin A1 band after PCR amplification 1000 bp; lane 2, amplified vector pET-28a^(+)^ 7000 bp; lane 3, recombinant Annexin A1- pET-28a^(+)^ band having total sequence 6376 bp.

**Figure 3:**
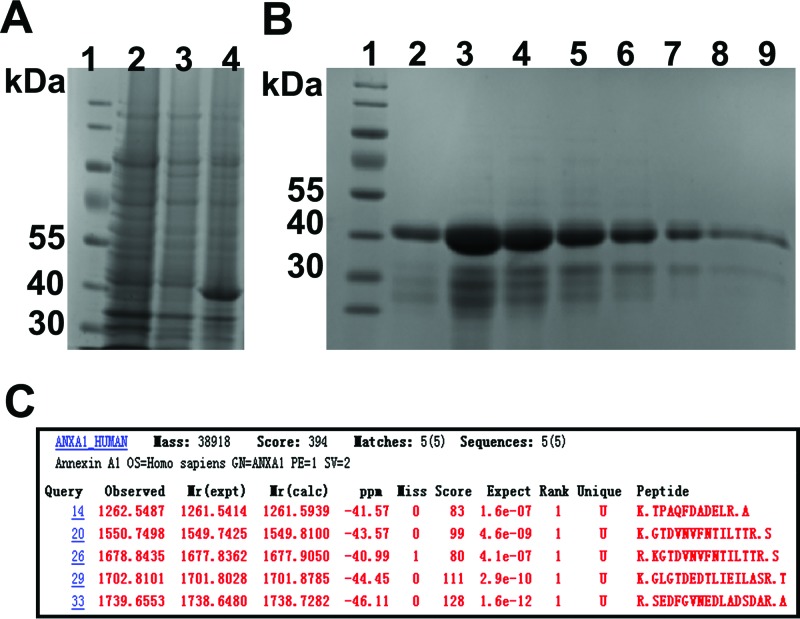
SDS/PAGE and MS (**A**) The IPTG-inducible expression of Annexin A1 in *E. coli* BL21 (DE3). Lane 1, protein marker; lanes 2 and 4, recombinant Annexin A1 with 0.1 mM IPTG-inducible expression in *E. coli* BL21; lane 3 without IPTG-inducible expression. (**B**) Protein purification by SDS/PAGE**:** recombinant Annexin A1 (39 kDa) electrophoresed in 12% acrylamide concentration (lanes 1–9). Lane 1, protein marker; lanes 2–9, purified recombinant Annexin A1, lanes 2 and 6–9 have high purity. (**C**) Mass spectrum results**:** MASCOT search Engine illustrates five matching sequences of peptides of Annexin A1.

After PCR amplification, 1% agarose gel electrophoresis was performed to separate the target molecule. Recombinant Annexin A1 protein band appeared at 1000 bp and no other specific amplifications appeared on the same gel as shown in Figure 2. The expression vector was constructed and validated by double restriction enzyme cleavage as shown in Figure 2. Then the recombinant product (vector product + *Annexin A1* gene) was analyzed by gel electrophoresis as mentioned in Figure 2. Recombinant *Annexin A1* gene was overexpressed as a fusion protein with N-terminal of hexahistidine-tag with/without induction of IPTG and was determined as shown in Figure 3A, then the product was purified with Ni-NTA resin kit and the presence of eluted target protein was confirmed by SDS/PAGE as shown in Figure 3B.

### MS analysis

Extracted Annexin A1 was reassured by MS. Mass spectrum identification showed that individual ions score is greater than 28, indicating that the putative protein has homologous identity or extensive homology and significant *P*-value (*P*<0.05). Protein scores are derived from ions scores as a nonprobabilistic basis for ranking protein hits as shown in Figure 3C.

### ELISA results

**Figure 4:**
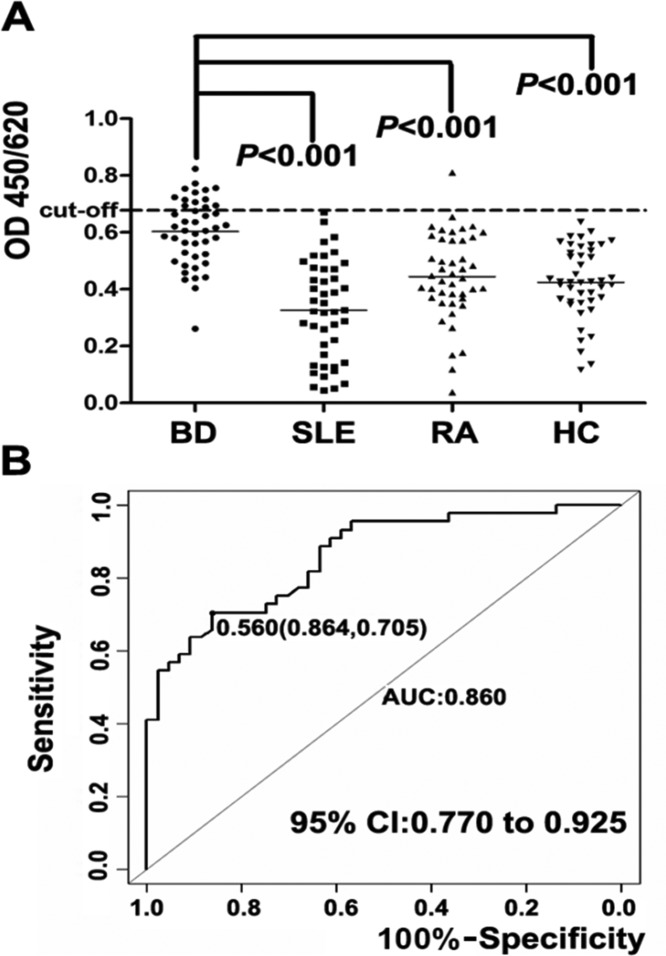
ELISA results (**A**) Scatter plot of ELISA results demonstrating the difference of antibodies between BD, SLE, RA, and HC. (**B**) ROC curve demonstrates specificity and sensitivity levels and 95% confidence intervals (CI).

To determine the titer of anti-Annexin A1 autoantibodies in BD patients, the reactivity of BD serum with recombinant Annexin A1 was performed by homemade ELISA. One hundred and seventy-six serum samples having 44 for each category (BD, SLE, RA, and HC) were analyzed with the same operating procedure. Initially these serum samples were tested with secondary antibodies conjugated with HRP (IgG, IgA, IgM). Only IgM secondary antibody showed elevated reaction with BD serum samples. In this test, anti-Annexin A1 autoantibodies were detected in 13 (29%) of 44 BD patients and 1 of 44 RA patients (<1%), while none of autoantibodies detected above the cut-off value in HC individuals as well as SLE patients. BD patients serum reaction against recombinant Annexin A1 with secondary antibody IgM was measured higher than HCs (*P*<0.001). Anti-Annexin A1 antibodies in all groups are described in a scatter plot which show clear differences between BD, SLE, RA, and HCs (*P*<0.0001) (Figure 4A).

### Statistical analysis

Fisher’s exact and Wilcox tests were applied by using SPSS software (version 21, Chicago, IL) and the results showed that *P*<0.05 was considered significant. The critical point for positive definition was a number with a higher value than that of the HCs (mean + 2 S.D.). MedCalc software was used to calculate the area under curve (AUC) which discriminate the BD from HC groups. Scatter plot showed significant results when compared BD patients with HCs (*P*<0.001). Cut-off value was 0.6783 at 100 specificity. Accuracy of the test was determined by the area under ROC curve (AUC =0.86) with confidence intervals (CI) =95%, which clearly classify the two groups (BD and HC) as depicted in Figure 4B.

## Discussion

Annexins are calcium and phospholipid-binding proteins and their family members expressed both in animals and plants [29]. Historically, Annexin A1 and A2 were first discovered in Rous sarcoma viruses major cellular substrates, which play a causative role in cancer development. Although, no human disease due to mutation in Annexins gene as a primary cause have been reported till now. But some studies revealed that changes in Annexins gene expression can be correlated with pathophysiology of diseases [30].

In present study, *Annexin A1* gene was cloned, purified, and identified as an autoantigen associated with BD. Using bioinformatics tools, we found that Annexin A1 and A2 have similar protein sequences and structural homology, and found sharing antigenic epitopes between them. Sequence similarity between two proteins was approximately 55% which indicates that both proteins have evolutionary relationship and certain numbers of the amino acid residues with conserved regions. The sequence similarity indicates that they have immunogenic coexistence. Similarly, the 3D structural comparison between two proteins (Annexin A1: 33–346 aa; Annexin A2: 1–339 aa) showed significant similar structural pairing with probability *P*<0.05 and raw score (score =927.07). Our results indicate that the level of expression in anti-Annexin A1 and A2 antibodies correlates and has similar antigenic worth. As an evidence, epitope spreading theory also states that those proteins, which have common epitopes, can cross-react with each other in the same protein and/or to other closely associated ones and these similar antigenic epitopes play an important role in autoimmune diseases [31].

Similarly, NielsKaj Jerne (Nobel Laureate) proposed that epitopes are cross-reactive. When antibodies recognize similar antigenic epitopes then they may cross-react with each other and form the immune complex and when their epitopes are exposed to other antibodies then it may become the new target of immune response. These theories support our findings and these autoantibodies (Annexin A1 and A2) may cross-react with each other in this way and might become a new immune target of BD.

**Figure 5:**
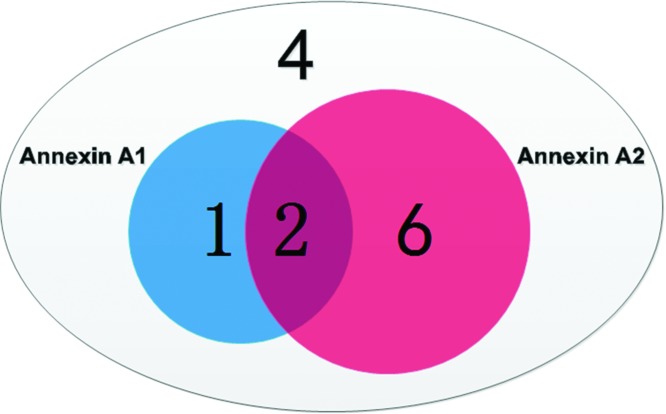
Comparative analysis of Annexin A1 and A2 reactivity with BD sera Positive rate of Annexin A1, 1/13, Annexin A2, 6/13, coexistence, 2/13, and 4 sera show negative reactivity with both proteins.

In this study, we also performed independent ELISA on 13 BD patients which showed the correlation between two autoantibodies as, 1 of 13 independently reacted with A1, 2 of 13 reacted with both A1 & A2, 6 of 13 independently reacted with A2 and 4 of 13 found non reactive, as depicted in Figure 5.

Although, we believed that Annexin A1 was not a candidate diagnostic biomarker in clinical application for all BD patients, but keeping in view the above facts and findings, the present study provides a new thinking of co-relationship between two autoantibodies belonging to the same family and their development mechanism in autoimmune diseases. However, we still have a long way to go to understand the precise functions of individual Annexins and their correlations in other autoimmune diseases. In the light of these findings, the author has confidently demonstrated the results and found a close association of Annexin A1 with Annexin A2 in patients with BD and it may be an autoantigen of BD which may be helpful in early and precise diagnosis of this disease.

## Abbreviations

AECA: anti-endothelial cell antibody
AUC: area under curve
BD: Behçet’s disease
FATCAT: flexible structure alignment by chaining aligned fragment pairs allowing twist
HC: healthy control
HUVEC: human umbilical vein endothelial cell
PBST: PBS solution with Tween-20
RA: rheumatoid arthritis
ROC: receiver operating characteristic
SLE: systemic lupus erythematosus
